# A new autosomal dominant eye and lung syndrome linked to mutations in *TIMP3* gene

**DOI:** 10.1038/srep32544

**Published:** 2016-09-07

**Authors:** Isabelle Meunier, Béatrice Bocquet, Gilles Labesse, Christina Zeitz, Sabine Defoort-Dhellemmes, Annie Lacroux, Martine Mauget-Faysse, Isabelle Drumare, Anne-Sophie Gamez, Cyril Mathieu, Virginie Marquette, Lola Sagot, Claire-Marie Dhaenens, Carl Arndt, Patrick Carroll, Martine Remy-Jardin, Salomon Yves Cohen, José-Alain Sahel, Bernard Puech, Isabelle Audo, Sarah Mrejen, Christian P. Hamel

**Affiliations:** 1 Institute for Neurosciences of Montpellier U1051, University of Montpellier-University Hospital, Genetics of Sensory Diseases, Montpellier, France; 2 Center for structural biochemistry Montpellier, INSERM U1054-CNRS UMR5048, Montpellier, France; 3 Sorborne Universités, UPMC Univ Paris 06, INSERM, CNRS, Institut de la Vision, 17 rue Moreau, 75012 Paris. CHNO des Quinze-Vingts, DHU Sight Restore, INSERM-DHOS CIC1423, 28 rue de Charenton, 75012 Paris. Institute of Ophthalmology, University College of London, London EC1V 9EL, UK; 4 Service d’Exploration de la Vision et Neuro-ophtalmologie, Hôpital Robert Salengro, CHU de Lille, France; 5 Fondation Adolphe de Rothschild, 25 rue Manin, 75019 Paris; 6 Département de pneumologie et d’addictologie. University Hospital Arnaud de Villeneuve, Montpellier, France; 7 Chest and Heart Imaging department, Arnaud de Villeneuve Hospital, Montpellier, France; 8 CHU Lille, Institut de Biochimie et Biologie Moléculaire, F-59000 Lille, France; 9 Eye clinic, Hôpital Robert Debré, CHRU de Reims, France; 10 Department of Thoracic Imaging, Hospital Calmette, University Centre of Lille, France; 11 Ophthalmic center for imaging and laser, rue Antoine Bourdelle, Paris - Department of Ophthalmology, Intercity Hospital and University Paris Est, Créteil, France

## Abstract

To revisit the autosomal dominant Sorsby fundus dystrophy (SFD) as a syndromic condition including late-onset pulmonary disease. We report clinical and imaging data of ten affected individuals from 2 unrelated families with SFD and carrying heterozygous *TIMP3* mutations (c.572A > G, p.Y191C, exon 5, in family 1 and c.113C > G, p.S38C, exon 1, in family 2). In family 1, all SFD patients older than 50 (two generations) had also a severe emphysema, despite no history of smoking or asthma. In the preceding generation, the mother died of pulmonary emphysema and she was blind after the age of 50. Her two great-grandsons (<20 years), had abnormal Bruch Membrane thickness, a sign of eye disease. In family 2, eye and lung diseases were also associated in two generations, both occurred later, and lung disease was moderate (bronchiectasis). This is the first report of a syndromic SFD in line with the mouse model uncovering the role of TIMP3 in human lung morphogenesis and functions. The *TIMP3* gene should be screened in familial pulmonary diseases with bronchiectasis, associated with a medical history of visual loss. In addition, SFD patients should be advised to avoid tobacco consumption, to practice sports, and to undergo regular pulmonary examinations.

Sorsby fundus dystrophy (SFD, MIM 136900) is a rare autosomal dominant retinal dystrophy described by Sorsby, Mason and Gardener in 1949^1^. SFD is characterized by bilateral deposition of yellow drusen-like material and extensive choroidal neovascularization (CNV) occurring between the third to fifth decades of life. Despite variable and confounding phenotypes, this dystrophy should be considered in patients with early neovascularization in absence of predisposing factors such as high myopia, or with irregular thickening of the Bruch membrane as shown on SD-OCT.

Weber and al. published in 1994 the first causal heterozygous mutation in the tissue inhibitor of metalloproteinases -3 (*TIMP3*, 22q12.3, 5 exons) in a family with SFD^2^. The autosomal dominant inheritance was further confirmed in all families, including those with a presumed recessive trait. TIMP3 as well as TIMP1 and TIMP2 regulate extracellular matrix renewal by the inhibition of matrix metalloproteinases (MMP). TIMPs form complexes with MMP-1, MMP-2, MMP-3, MMP-7, MMP-9, MMP-13, MMP-14 and MMP-15 and irreversibly inactivate them by binding to their catalytic zinc cofactor. The NH2-terminal domain of TIMP1, TIMP2 and TIMP3 is responsible for MMP inhibition, whereas the COOH-terminal domain is involved in additional functions of the protein. In the retina, TIMP3 interacts with epithelial growth factor-containing fibulin-like extracellular matrix protein 1 (EFEMP1) and blocks the binding of VEGF to VEGFR2 resulting in inhibition of angiogenesis^3^,^4^,^5^,^6^,^7^,^8^,^9^,^10^,^11^,^12^,^13^. The majority of known mutations in *TIMP3* underlying SFD are located in exon 5, with a substitution by a cysteine residue in most cases (for examples codons 174, 177 and 191). In the eye, Sorsby fundus retinal dystrophy is then characterized by a thickening of Bruch Membrane and subsequent risk of choroidal neovascularization after the age of 40. The thickening of Bruch Membrane in SFD is located between the basement membrane of the RPE and the inner collagenous zone as previously reported in electron microscopic studies^14^,^15^. The current pathological hypothesis is that TIMP3 mutants with unpaired cysteine residue lead to the formation and accumulation of dimers in the extracellular matrix (ECM, Bruch Membrane). Bruch Membrane thickening contributes to a relative hypoxia and a dysfunction of the RPE with occurrence of choroidal neovascularization and progressive RPE loss.

Chronic Obstructive Pulmonary Disease (COPD) is characterized by an irreversible airflow limitation, caused by an increase in the resistance of the small conducting airways and in lung compliance due to emphysema^16^,^17^,^18^,^19^,^20^. The main causes are tobacco smoking together with atmospheric and domestic pollution. As only 15 to 20% smokers develop the disease, genetic factors could be involved in the pathogenesis. To date, polymorphisms in metalloproteinases (MMP3, MMP9) and their inhibitors (TIMP2, TIMP3) have been shown to be implicated^21^,^22^.

So far, SFD was strictly considered as an ocular disease. We report herein for the first time two unrelated families carrying heterozygous mutations in *TIMP3* with an autosomal dominant syndromic form of SFD associated with pulmonary disease.

## Results

### Clinical findings

All SFD patients older than 55 years had pulmonary involvement either moderate (asymptomatic air trapping) or severe (severe panlobular emphysema, very severe obstruction and chronic respiratory failure).

#### Family 1

##### Ocular involvement

**Figure 1:**
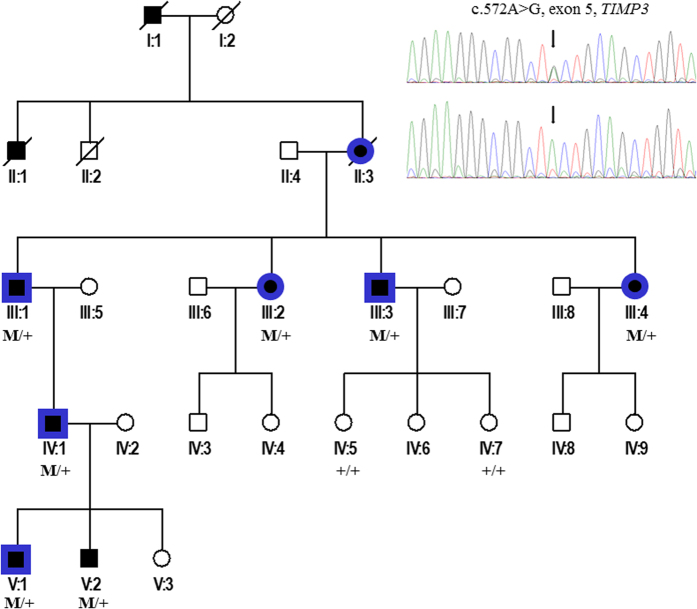
Family 1 with SFD and severe pulmonary disease linked to c.572A  > G *TIMP3* mutation (squares = men and circles = women, black = SFD, blue = pulmonary disease, M/+: affected patients and +/+: wild type).

**Figure 2:**
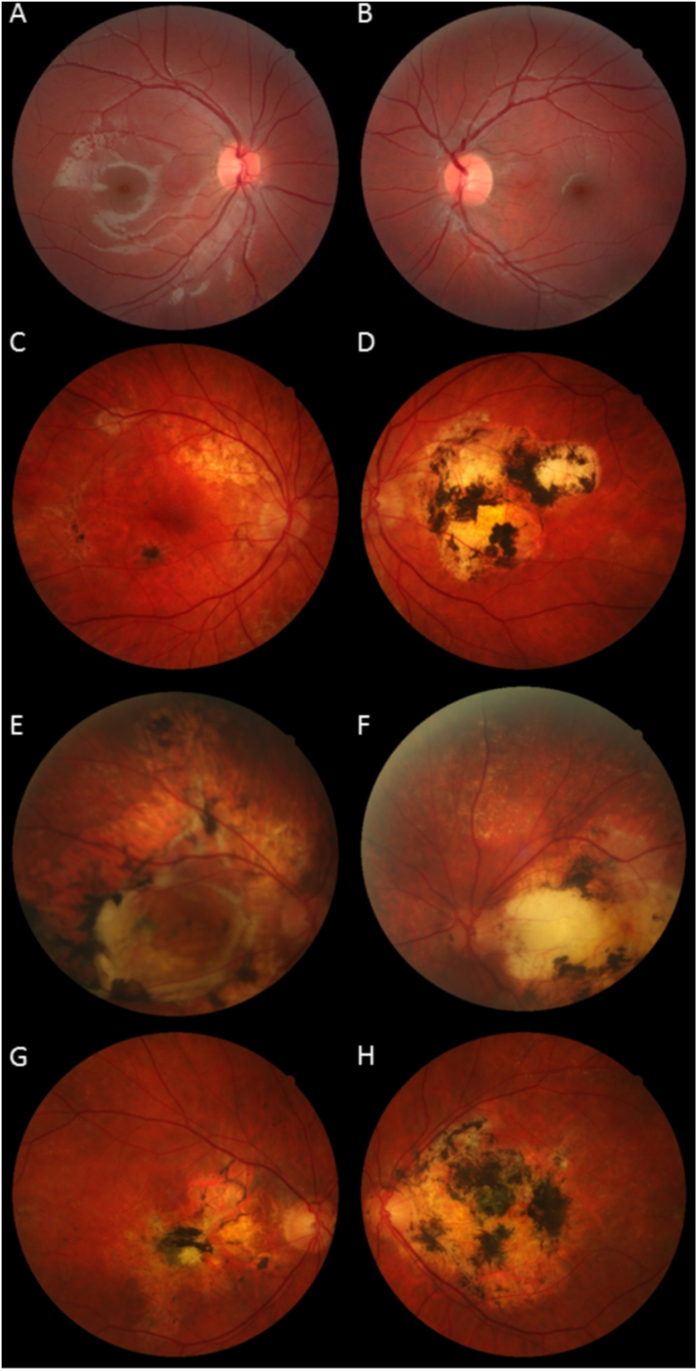
Family 1. Color fundus photographs. (**A**,**B**) The youngest patient (V:2 ) had a normal fundus appearance. His father (**C**,**D**- IV:1) had a severe macular scarring in his left eye (**D**) and a preserved macula in his right eye by the use of anti-VEGF injections (**C**). Patients III:3 (**E**,**F**) and III:4 (**G**,**H**) had severe macular lesions.

In this family (Figs 1 and 2), the 5 patients older than 30 had extensive bilateral CNV with diffuse drusen-like changes (Table 1). CNV occurred at a young age (23 to 40 years). The oldest patient complained of moderate night-blindness. All were legally blind except patient IV:1, who received intravitreal injections of anti-vascular endothelial growth factor (bevacizumab) and retained 20/20 best-corrected visual acuity in the right eye. The grand-mother (II:3) of patient IV:1 was legally blind after the age of 50. The two younger boys (V:1, V:2) were asymptomatic, but their Bruch Membrane was abnormally thickened on SD-OCT.

##### Pulmonary involvement

**Figure 3:**
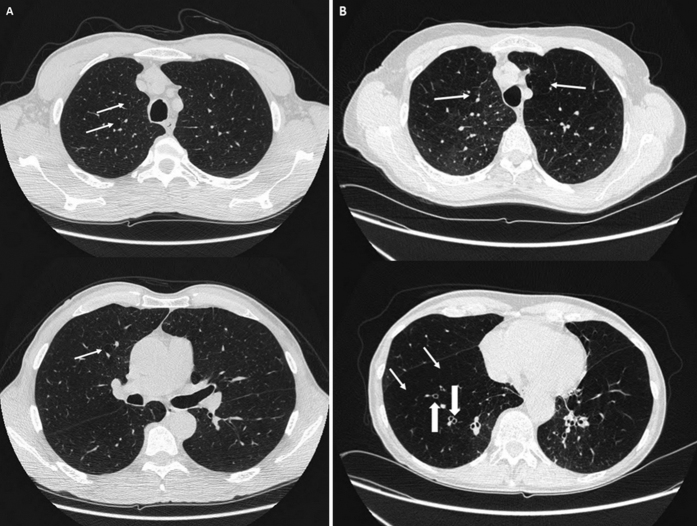
High resolution computed tomographic scans of patients III:3 (**A**) and III:2 (**B**). On CT scans, note bronchiectasis, diffuse bronchial wall thickening (large arrows), air trapping and emphysema bullae (thin arrows). These lesions are localized at the basal part of the lung, and not at the apex as noted in smokers. Patient III:2 uses domiciliary oxygen.

Without smoking exposure or any other pulmonary disease, generation III (70, 67, 59 and 53 years) had airflow limitation with a reduction of (FEV1) between 28% and 73% (according to American and European Thoracic Society criteria). On CT scans, these patients showed air trapping and abnormal bronchial wall thickening with mild basal bronchiectasis (Fig. 3). Two of them (III:1 and III:2)had chronic respiratory failure with oxygen supplementation. Their mother (II:3) died from severe emphysema. Patients IV:1 (44 years) had air trapping on chest tomography with normal pulmonary function tests. Patient V:1 (16 years) had air trapping and hypoxemia (79 mmHg) at rest. This patient had also complex cardiac congenital malformation with ventricular septal defect, obstruction of the right ventricular outflow tract, mitral valve dysplasia and hypoplasia of the left ventricle. An emergency surgical procedure was performed at 15 days of life for cyanosis. His brother V:2 (14 years) had a normal thoracic tomography and normal pulmonary function tests at rest.

#### Family 2

##### Ocular involvement

**Figure 4:**
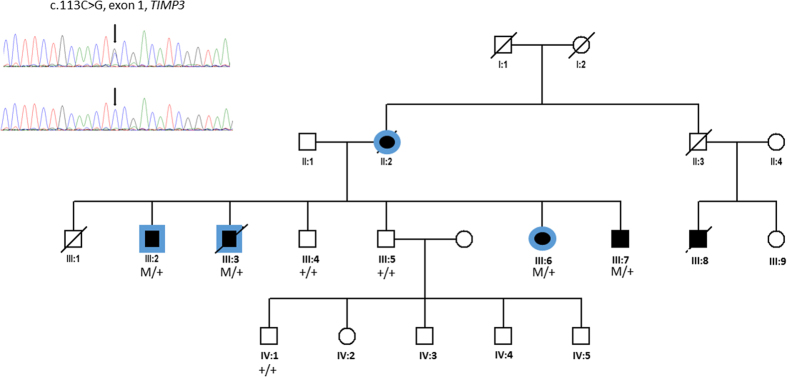
Pedigree of family 2 with SFD and late moderate pulmonary disease linked to c.113C > G *TIMP3* mutation. (squares = men, circles women, black = SFD and blue = pulmonary disease, M/+: affected patients and +/+: wild type).

**Figure 5:**
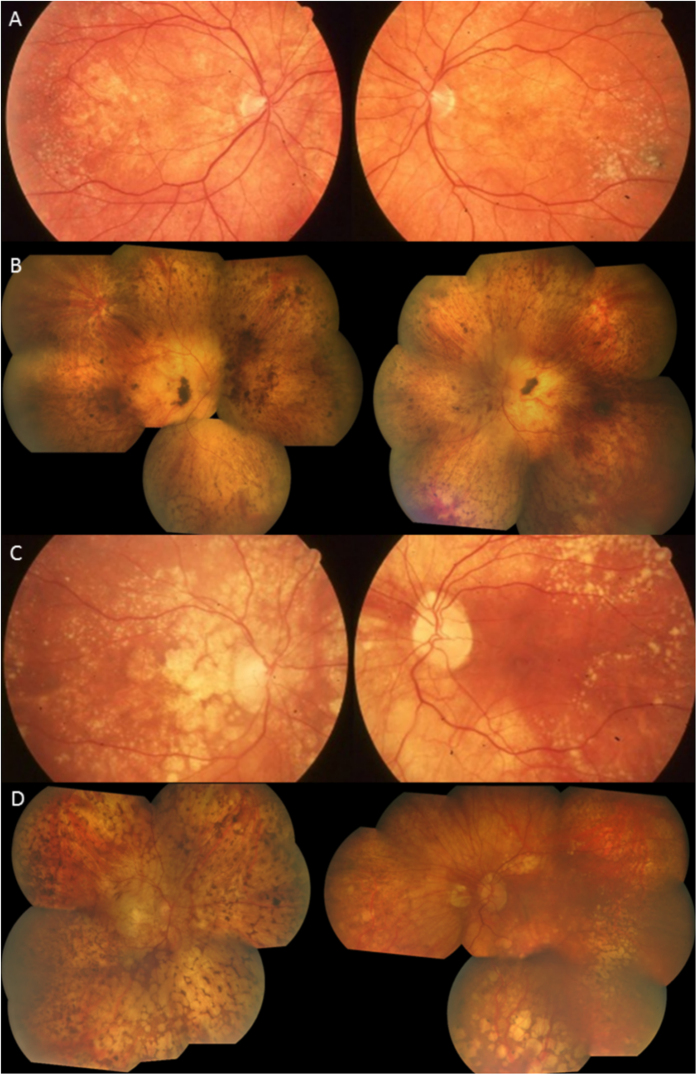
Color fundus photographs. (**A**) Patient III:2 at the age of 55, note the numerous drusen-like lesions and yellow large material all over the retina. (**B**) Same patient at the age of 81, macular atrophy occurred with subsequent ambulatory vision loss. (**C**) Patient III:6 at the age of 57, note the atrophic lesions at the posterior pole in the right eye. (**D**) Same patient at the age of 65.

Night blindness, reported after the age of 45 was the first symptom in this family (Figs 4 and 5) (Table 1). At onset, multiple diffuse drusen-like accumulations or large yellow lesions were observed at the posterior pole and in the whole peripheral retina. In patients III:2- 3 and 6, after the age of 40 to 50 years, best-corrected visual acuity decreased rapidly in both eyes in less than 10 years due to recurrent and extensive choroidal neovascularization. At the final stage, extensive subretinal fibrosis and macular atrophy with pigmentary changes were observed in both eyes (Fig. 5). The mother (patient II:2) carried the presumed diagnosis of macular degeneration and was blind before 60 years.

##### Pulmonary involvement

**Figure 6:**
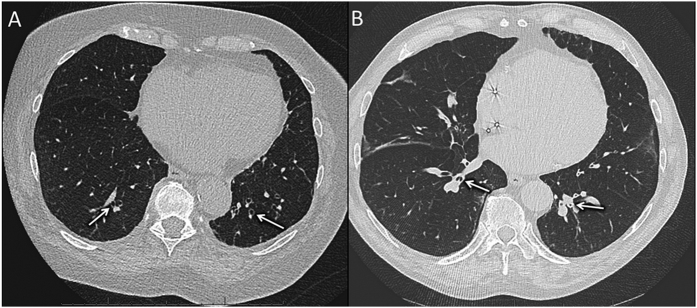
High resolution CT scan. (**A**) Patient III:6, note mild cylindered bronchiectasis in both lower lobes (white arrows). (**B**) Patient III:2, there is marked bronchial wall thickening on both sides (whites arrows).

In this family, the two living patients of generation III underwent chest CT and had mild to marked bronchial wall thickening with mild cylindrical bronchiectasis in a single case (Fig. 6). None had CT features of emphysema. The mother II:2 developed after the age of 70 years atypical asthma with no history of smoking.

### Genetic findings

#### Family 1

We found the *TIMP3* variation c.572A > G (p.Y191C) in exon 5, previously described in a family with isolated SFD (initially p.Y168C)^2^. This variation is considered to be probably damaging by PolyPhen 2 with a score = 1 (http://genetics.bwh.harvard.edu/pph2), damaging according to SIFT with a score = 0 (http://sift.jcvi.org), and to interfere most likely with the function of the protein by the align-GVDG program with a class C65 (http://agvgd.iarc.fr/agvgd_input.php), disease causing for mutation taster with a probability of 0.99 (www.mutationtaster.org), and neutral for Provean (provean.jcvi.org). The mutation co-segregated with the disease in the family and is not reported in the ExAc database (http://exac.broadinstitute.org).

Multiple amino-acid sequence alignment of TIMP3 orthologs showed the conservation of the leucine at position 191 in all eukaryotic sequences available. According to Nextprot database (www.nextprot.org), this mutation has been noted in several families since the initial identification of *TIMP3*^2^,^23^.

#### Family 2

We found the *TIMP3* variation c.113C > G (p.S38C) in exon 1 which has been previously described in SFD^24^. This variation is considered to be probably damaging by PolyPhen 2 with a score = 1 (http://genetics.bwh.harvard.edu/pph2), damaging according to SIFT with a score = 0 (http://sift.jcvi.org), and to interfere most likely with the function of the protein by the align-GVDG program with a class C65 (http://agvgd.iarc.fr/agvgd_input.php), disease causing for mutation taster with a probability of 0.99 (www.mutationtaster.org), and deleterious for Provean (provean.jcvi.org). This variation is not present in ExAC. In addition, the mutation co-segregated with the disease in the family. This mutation has been reported previously in 5 SFD unrelated patients^24^,^25^.

## Discussion

This is the first report of a syndromic association of autosomal dominant SFD with an autosomal dominant pulmonary disease. This latter aspect is characterized by distal bronchiolar and alveolar dysfunctions leading to a chronic obstructive pulmonary disease in absence of asthma and tobacco consumption. In addition, the basal location of pulmonary lesions is not in keeping with COPD secondary to smoking. In these families, the two oldest generations developed pulmonary lesions in line with an autosomal dominant transmission and with an extracellular matrix remodeling disease (Bruch Membrane in the eye). Indeed, there are several experimental data that plead for the pathological role of TIMP3 mutations in the pulmonary disease.

First, TIMP3 is highly expressed in the lung. Its role in this organ was well established in developmental phases, in resolution of inflammation following lung injury and in idiopathic pulmonary fibrosis^26^,^27^,^28^. At the developmental stage, bronchiole branching morphogenesis depends on interactions between the bronchiole epithelium, the mesenchyme and the extracellular matrix or basement membrane which serves as an interface between the two compartments. Remarkably, it was shown that in Timp3-/- null mice, enhanced MMP activity interferes with extra cellular matrix proteolysis, perturbing the formation of the bronchiole tree during morphogenesis. These homozygous mice have abnormal bronchiolar morphology and respiratory dysfunction with reduction in carbon monoxide uptake and a progressive increased alveolar size proved by a decline in hydroxy-proline content (http://www.informatics.jax.org/allele/genoview/MGI:3056101).

Another aspect of the role of TIMP3 in the pulmonary disease is its involvement in the resolution of inflammation following lung injury, by regulating the neutrophil influx in the injury site. In Timp3-/- mice, the inflammation persisted up to 28 days with increased neutrophil chemotactic activity^28^. This prolonged abnormal response was reversed under synthetic inhibitor of MMP. In addition, in idiopathic pulmonary fibrosis characterized by fibroblast expansion and extracellular matrix accumulation, TIMP3 gene expression is increased and the protein is localized to fibroblastic foci and extracellular matrix. This dysregulation of ECM remodeling could involve in the lung the p38 kinase pathway and the TGF-beta1 which are important mediators in lung fibrosis.

The role of polymorphisms in metalloproteinases (MMP3-MMP9, ADAM33) and their inhibitors (TIMP2 and TIMP3) in the onset and severity of COPD in smokers has been reported^21^,^22^. In our families, a mutation in TIMP3 is also probably the cause of the autosomal dominant lung disease with lung extracellular matrix damaging.

This syndromic disease could be unsuspected because the pulmonary disease starts one to three decades after visual loss. For example, in our family 2, pulmonary involvement was only confirmed by CT imaging. This new syndrome has an autosomal dominant inheritance as it concerns three generations (6 patients) in family one and two generations (three patients) in family two.

**Figure 7:**
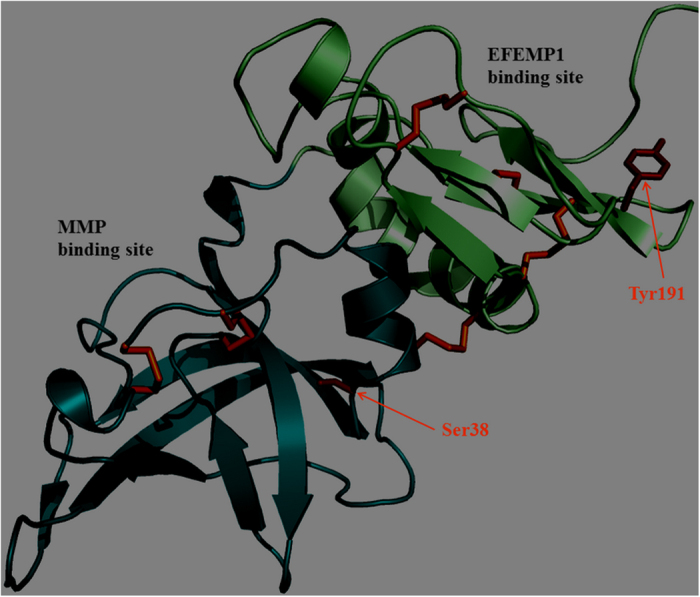
Representation of a theoretical model of the TIMP3 mature protein. The main-chain is shown as ribbon with the N-terminal domain (24–127) and C-terminal domain (128–209) colored in light blue and light green, respectively. The side-chain of the cysteines and the two mutated residues (S38 and Y191) are shown as stick in orange and red color, respectively. The figure was prepared using Pymol (http://www.pymol.org).

To shed light on the potential impact of the two mutations and differences of disease onset or severity, a theoretical model of the full-length TIMP3 was based on the crystal structure of TIMP2 and TIMP3 as a combined template using the server @TOME-2 (Pons et Labesse 2009)^29^,^30^. The differences in lung severity involvement between families 1 and 2 could be linked with both the type of substitutions/mutations (non-conservative/conservative, respectively) and their locations in distinct domains (C-terminal and N-terminal respectively, Fig. 7). Indeed, in family 1, a large and hydrophobic residue is substituted by a small cysteine while in the family 2, a serine is substituted by an isosteric cysteine. Furthermore, in the first case, the additional cysteine lies spatially close to two disulphide bridges (C145–C192; C163, C184) and it may interfere with them especially during TIMP3 folding and maturation. In the second case, the substitution occurs further from any other cysteines (C36–C143). Finally, the two mutated sites correspond to two distinct protein-protein interaction sites. The mutation p.S38C is located at the interface with the MMPs and is not predicted to affect significantly the binding affinity. On the contrary, due to a more pronounced change in amino-acid size, the mutation p.Y191C could impact more dramatically on the interaction with EFEMP1 which has been mapped to the C-terminal part of TIMP3^13^. Accordingly, the mutation in family 1 is predicted to be more detrimental to the protein function and/or stability than the second mutation. It should be noted that EFEMP1 is also expressed in the lung, but we cannot exclude that TIMP3 interacts with a distinct lung specific molecule. Regarding SFD, CNV occurred during the third and fourth decades in family 1 versus the fifth decade for family 2. The other reported cases with p.S38C and p.Y191C displayed similar differences in age of onset^2^,^23^,^24^,^25^.

In conclusion, Sorsby fundus dystrophy should be reappraised as a syndromic condition with a risk of late onset bronchiolar and pulmonary disease. SFD patients should avoid tobacco smoking and practice sports. Furthermore, a pulmonary disease should be investigated after the age of 55. In this line, *TIMP3* should be screened in patients with familial bronchiectasis or emphysema, particularly if a medical history of visual loss or choroidal neovascularization is reported.

## Patients and Methods

Informed consent was obtained for clinical examination and genetic analysis from all patients. All methods were carried out in accordance with approved protocols of Montpellier and Lille University Hospitals, and in agreement with the Declaration of Helsinki. The Ministry of Public Health accorded approval for biomedical research under the authorization number 11018S.

### Clinical and functional retinal evaluation

For each patient, age at examination, refraction, initial and final best-corrected visual acuity were noted. The best-corrected visual acuity was obtained with Snellen charts. Near visual acuity was assessed with the current French near vision chart (Parinaud). Color fundus photographs were performed with Topcon Imagenet (Ophthalmic Imaging Systems, Japan) or Nidek non-mydriatic automated fundus camera AFC 330 (Nidek Inc, Japan). Autofluorescence imaging and spectral domain optical coherent tomography were performed with Combined Heidelberg Retina Angiograph + OCT Spectralis device (Heidelberg Engineering, Dossenheim, Germany).

### Pulmonary evaluation

#### Chest computed tomography (CT)

We performed high-resolution chest CT scans in 8 patients (6 in family 1 and 2 in family 2). Chest CT was obtained on different multi-detector (MDCT) scanners, including a 64-slice MDCT equipment (SOMATON Definition AS+, SIEMENS, Healthcare, Forchheim, Germany) and a third-generation, dual source CT scanner (SOMATOM Force, SIEMENS Healthcare, Forchheim, Germany) detectors. The scanning protocol included end-inspiratory and -expiratory acquisitions over the entire thorax. The CT parameters analyzed on lung images included emphysema (i.e., centrilobular, panlobular, bullous), bronchial wall thickening, bronchiectasis and CT features of small airways disease (i.e., bronchiolectases, ill-defined micronodules, mosaic attenuation and air trapping) on lung images.

#### Pulmonary function test

Spirometry was performed in all affected patients of family 1, in accordance with American Thoracic Society standards^19^,^31^. Values of percent-predicted for spirometry were calculated using reference values based on age, height, sex and race^19^,^31^. The basic parameters used to properly interpret lung function were forced vital capacity (FVC), forced expiratory volume in 1 s (FEV1), and FEV1/FVC ratio. Airway obstruction was defined by reduction of the FEV1/FVC ratio (also known as Tiffeneau-index) below 70%.

### Genetic analysis

Genomic DNA was extracted using a standard salting-out procedure. All exons of *TIMP3* (refseq NG_009117.1) were screened in all patients. The screening was performed on genomic DNA using primers designed to flank the splice junctions of each exon (sequences available upon request). After standard polymerase chain reaction (PCR) amplification, products were purified with ExoSAP-IT (GE Healthcare Life Sciences, USB Corporation) and direct sequencing was performed on an Applied Biosystems (ABI) 3130 xL genetic analyzer (Applied BioSystems, Foster City, CA, USA) using the BigDye Terminator Cycle Sequencing Ready Reaction kit V3.1. Sample sequences were aligned to the wild-type sequence and analyzed with the Collection and Sequence Analysis software package (Applied Biosystems).

The pathogenicity of nucleotide changes was estimated by different predictive software including, Polyphen program - Harvard University, Boston, MA. http://genetics.bwh.harvard.edu/pph, SIFT (http://sift.jcvi.org), Align-GVDG program (http://agvgd.iarc.fr/agvgd_input.php), Mutation taster (www.mutationtaster.org), and Provean (provean.jcvi.org). The pathogenicity was also assessed considering multiple-amino-acid sequence alignment of *TIMP3* orthologs and the protein structure identity according to Nextprot database (www.nextprot.org). Indeed, the variation had to segregate with the disease in the family. The identified variations were tracked in all genetic databases and in previous articles about Sorsby fundus dystophy.

## Additional Information

**How to cite this article**: Meunier, I. *et al*. A new autosomal dominant eye and lung syndrome linked to mutations in *TIMP3* gene. *Sci. Rep.* **6**, 32544; doi: 10.1038/srep32544 (2016).

## Figures and Tables

**Table 1:** Clinical data of patients with Sorsby fundus dystrophy.

Patient	Gender	Onset	Age at examination	Visual acuity at Examination	Age at CNV occurrence	Chest computed tomography
Family 1: Mutation in *TIMP3*, exon 5, c.Y191C						
III:1	M	40	65	RE: CF	RE: 40	Severe emphysema
				LE: CF	LE: 42	
III:2	F	39	68	RE: CF	RE: 39	Severe emphysema
				LE: CF	LE: 43	
III:3	M	34	60	RE: CF	RE: 34	Mild emphysema
				LE: 20/400	LE: 40	
III:4	F	29	54	RE:20/200	RE: 29	Mild emphysema
				LE:20/200	LE: 33	
IV:1	M	23	45	RE:20/20	RE: 35	Air trapping
				LE:20/200	LE: 23	
V:1	M	asymptomatic	16	RE:20/20	No CNV	Air trapping (cardiac malformation)
				LE:20/20	RE-LE	
V:2	M	asymptomatic	14	RE:20/20	No CNV	Normal imaging
				LE:20/200	RE-LE	
Family 2 Mutation in *TIMP3*, exon 1, c.S38C						
III:2	M	50	72	RE: CF	No CNV	Air trapping Bronchiectasis
				LE: CF	RE-LE	
III:3	M	40	deceased	RE: CF	RE: 40	Not practiced Deceased
				LE: CF	LE: No CNV	
III:6	F	49	73	RE: CF	No CNV	Air trapping Bronchiectasis
				LE: CF	RE-LE	

M: male. F: female. Onset: age of onset i.e., symptomatic stage. Age: age at examination. RE: right eye. LE: left eye. CNV: choroidal neovascularization. CF: counting fingers.
